# Intrinsic Mechanism of CaCl_2_ Alleviation of H_2_O_2_ Inhibition of Pea Primary Root Gravitropism

**DOI:** 10.3390/ijms25168613

**Published:** 2024-08-07

**Authors:** Ruonan Wei, Lei Ma, Shaoying Ma, Ling Xu, Tingfeng Ma, Yantong Ma, Zhen Cheng, Junhong Dang, Sheng Li, Qiang Chai

**Affiliations:** 1 College of Life Sciences and Technology, Gansu Agricultural University, Lanzhou 730070, China; weirn@st.gsau.edu.cn (R.W.);; 2 Agronomy College, Gansu Agricultural University, Lanzhou 730070, China; 3 Laboratory and Site Management Center, Gansu Agricultural University, Lanzhou 730070, China; mashy@gsau.edu.cn; 4 State Key Laboratory of Arid-land Crop Science, Gansu Agricultural University, Lanzhou 730070, China

**Keywords:** primary root, gravitropism, H_2_O_2_, CaCl_2_, *Pisum sativum*

## Abstract

Normal root growth is essential for the plant uptake of soil nutrients and water. However, exogenous H_2_O_2_ inhibits the gravitropic growth of pea primary roots. It has been shown that CaCl_2_ application can alleviate H_2_O_2_ inhibition, but the exact alleviation mechanism is not clear. Therefore, the present study was carried out by combining the transcriptome and metabolome with a view to investigate in depth the mechanism of action of exogenous CaCl_2_ to alleviate the inhibition of pea primordial root gravitropism by H_2_O_2_. The results showed that the addition of CaCl_2_ (10 mmol·L^−1^) under H_2_O_2_ stress (150 mmol·L^−1^) significantly increased the H_2_O_2_ and starch content, decreased peroxidase (POD) activity, and reduced the accumulation of sugar metabolites and lignin in pea primary roots. Down-regulated genes regulating peroxidase, respiratory burst oxidase, and lignin synthesis up-regulated *PGM1*, a key gene for starch synthesis, and activated the calcium and phytohormone signaling pathways. In summary, 10 mmol·L^−1^ CaCl_2_ could alleviate H_2_O_2_ stress by modulating the oxidative stress response, signal transduction, and starch and lignin accumulation within pea primary roots, thereby promoting root gravitropism. This provides new insights into the mechanism by which CaCl_2_ promotes the gravitropism of pea primary roots under H_2_O_2_ treatment.

## 1. Introduction

Pea (*Pisum sativum* L.) is a strategic crop that ensures global food security and is favored for its versatility, nutrient richness, and adaptability [1]. The seedling stage of pea plants usually occurs during the low-rainfall season; thus, drought affects the growth of pea seedlings and, consequently, their yield and quality [2]. Roots absorb nutrients and water directly into the soil and act as sensors to sense and respond to a variety of external stresses [3]. As the only source of water and nutrient uptake for the seedling, the primary root must enter the soil without other support in order to be better adapted to the living environment [4]. Therefore, improving root growth capacity is a strategy to increase pea yield. It has been found that positive root geotropism is necessary for plant access to nutrients and water [5]. However, exogenous signals can change the plant’s root system architecture (RSA) and root growth direction, thus inhibiting plant growth [6]. Therefore, it is important to study the pathways that regulate the alteration of RSA and root growth direction.

Reactive oxygen species (ROS) are not only components that induce oxidative damage in the plant body but also act as signaling molecules to regulate plant growth [7]. ROS can alter the RSA by regulating lateral root formation in *Arabidopsis thaliana* [8]. In contrast, intracellular ROS homeostasis is regulated by a series of enzymes that include antioxidant enzymes such as NADPH/NADH, superoxide dismutase (SOD) (EC 1.15.1.1), oxidases, and peroxidase (POD) (EC 1.11.1.7) [9,10]. It has been found that ROS are involved in the signaling of the plant hormones abscisic acid (ABA) and auxin (IAA), which regulate seed germination, root growth, and differentiation [11]. H_2_O_2_ mediates NADPH oxidase to enable ABA activation of lateral root development, the synthesis of H_2_O_2_, and cell wall expansion [12,13,14]. Interaction between ROS and IAA can regulate root geotropism, lateral and adventitious root formation, and lignification [15]. Synergistic interactions occur between IAA and gibberellin (GA), wherein IAA induces the degradation of the DELLA protein, which inhibits GA signaling and is involved in the promotion of GA biosynthesis gene expression in terms of root elongation and root division [16].

In addition, H_2_O_2_ is involved in the regulation of calcium signaling pathways. It has been found that the H_2_O_2_ produced by tobacco cell inducers may activate H_2_O_2_-sensitive Ca^2+^ channels in the plasma membrane, resulting in an increase in cytoplasmic Ca^2+^ concentrations [17]. Ca^2+^ channels can be activated by NADPH oxidase to regulate plant cell expansion and, thus, cell development [18]. External signals stimulate the amyloplasts to settle on the endoplasmic reticulum, causing Ca^2+^ to be effluxed into the cytoplasm. This may cause a transient increase in cytoplasmic Ca^2+^ concentration, which, upon binding to calmodulin, either directly or indirectly activates the downstream signal transduction mechanisms [19]. Whereas starch is a key substance in the perception of gravity by the root system [20], changes in Ca^2+^ concentration caused by starch deposition regulate the direction of root growth.

It was found that cell wall accumulation limits cell elongation, thereby altering the RSA [21]. Cell wall metabolism is a key factor in plant response to environmental stresses and it is mediated by a variety of cell wall-modifying proteins. Soluble arabinogalactan proteins (AGPs) and insoluble extensions (EXTs) are associated with each other in the cell wall, and the EXT/AGP complex acts on the structure of the cell wall to regulate changes in the shape of the cell wall [22,23]. Increased H^+^ concentrations activate extensin activity and activate the cell wall, which leads to cell elongation [24]. Therefore, it is important to investigate the mechanism of action of exogenous H_2_O_2_ and CaCl_2_ on the cell walls of the primary roots of pea plants.

From the above, it is clear that H_2_O_2_ and Ca^2+^ are key signaling molecules that regulate the gravity-oriented nature of plants. It has been shown that exogenous H_2_O_2_ inhibits the gravity-oriented force of pea primary roots, while CaCl_2_ can alleviate this inhibition to a certain extent, but the specific alleviation mechanism has not been clarified [25]. Therefore, it is crucial to study in depth the alleviating effect of CaCl_2_ on H_2_O_2_ stress. Thus, this study combined transcriptomics and metabolomics to comprehensively analyze the pathways regulating exogenous CaCl_2_ to alleviate the inhibition of pea root gravitropism by H_2_O_2_. We also verified the roles of key metabolites and genes by combining the relevant physiological indicators with fluorescence quantification. This study provides a theoretical basis for the gravity-oriented nature of plant roots in adversity.

## 2. Results

### 2.1. Root Non-Geostrophic Validation Experiments

In this study, we counted the bending rate (Table S1 and Figure S1 in the Supplementary Materials) and bending degree (Table S2 and Figure S2 in the Supplementary Materials) of pea primordial roots after 24, 36, 54, and 72 h of different treatments. The results showed that the shorter the treatment time, the greater the difference between replicates. Therefore, the main discussion in Section 3 is on the changes seen in the growth of pea primary roots at 72 h and the intrinsic mechanism.

**Figure 1:**
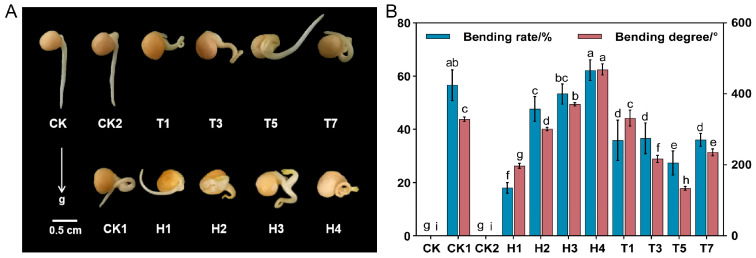
Effect of H_2_O_2_ and CaCl_2_ on the growth of pea primary roots. (**A**) Phenotypes of pea primary root growth under different treatments at 72 h. (**B**) Bending rate and bending degree of pea primordial roots under CaCl_2_-mitigated H_2_O_2_ treatment at 72 h. The growth phenotypes of pea primordial roots under different treatments at 72 h were as shown. Data are the mean ± SD of three replicates, and the error bars indicate the standard deviation of three replicates. Different lower-case letters are the results of significance analyses of Duncan’s multiple range test, indicating statistically significant differences (*p* < 0.05). CK: deionized water; CK1: 150 mmol·L^−1^ H_2_O_2_; CK2: 10 mmol·L^−1^ CaCl_2_; H1/H2/H3/H4: 20/80/200/300 mmol·L^−1^; T1/T3/T5/T7: 150 mmol·L^−1^ H_2_O_2_ + 1/5/10/15 mmol·L^−1^ CaCl_2_.

Pea primary roots grew curved under exogenous H_2_O_2_ treatment (Figure 1A), and the bending rate and bending degree of primary roots (Figure 1B and Table S3) gradually increased with increasing concentration. The bending rate of primary roots under 150 mmol·L^−1^ H_2_O_2_ treatment was 3.3-fold higher than that of 20 mmol·L^−1^ H_2_O_2_. The subsequent application of different concentrations of CaCl_2_ on top of the 150 mmol·L^−1^ H_2_O_2_ treatment (Figure 1) revealed that CaCl_2_ had a mitigating effect on the bending growth of pea primordial roots under the H_2_O_2_ treatment (Figure 1A). With increasing CaCl_2_ concentration, the bending rate and bending degree of primordial roots had minimum values at the 10 mmol·L−1 CaCl_2_ and were significantly lower than for the 150 mmol·L^−1^ H_2_O_2_ treatment (Table S3). The application of 10 mmol·L^−1^ CaCl_2_ to pea primordial roots alone revealed no significant difference in growth status with CK treatment.

To further verify the important role of Ca^2+^ in mitigating the H_2_O_2_ inhibition of root growth toward gravitropism, CaSO_4_ and KCl, used at the same ionic (Ca^2+^, Cl^−^) concentrations as the CaCl_2_ solution, were applied exogenously (Table S4 and Figure S3). It was found that the pea bending rate (Table S1 and Figure S1) and bending degree (Table S2 and Figure S2) were significantly reduced under the CaSO_4_ treatment compared with the 150 mmol·L−1 H_2_O_2_ treatment, and that these reached a minimum at a Ca^2+^ concentration of 10 mmol·L−1, while KCl treatment had no significant mitigating effect on H_2_O_2_ inhibition.

Based on these statistical results, pea primary roots under the 150 mmol·L−1 H_2_O_2_ and 150 mmol·L−1 H_2_O_2_ + 10 mmol·L−1 CaCl_2_ treatments were selected for the study of transcriptome and metabolome indexes.

### 2.2. Transcriptome Analysis and Validation of Key DGEs in Pea Primary Roots under H_2_O_2_ and CaCl_2_ Treatment

**Figure 2:**
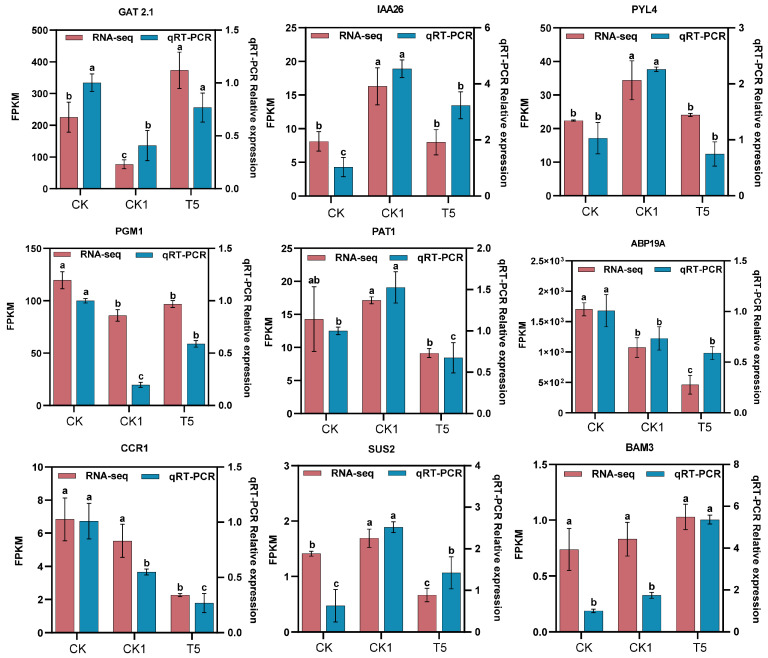
qRT-PCR analysis of DEGs in the primary roots under H_2_O_2_ and CaCl_2_ treatments. We selected nine DEGs regulating key metabolic pathways for qRT-PCR validation. The qRT-PCR values were compared with gene FPKM values to validate the reliability of the transcriptomic data. Different lower-case letters are the results of significance analyses of Duncan’s multiple range test, indicating statistically significant differences (*p* < 0.05). What is compared here is the significance of the same indicator between different treatments. Genes included *GAT2.1*, *IAA26*, *PYL4*, *PGM1*, *PAT1*, *ABP19A*, *CCR1*, *SUS2*, and *BAM3*. CK: water only, CK1: 150 mmol·L^−1^ H_2_O_2_, and T5: mmol·L^−1^ H_2_O_2_ + 10 mmol·L^−1^ CaCl_2_; the same apply in the figures below.

To investigate the mechanism of action of pea root growth under the CaCl_2_ mitigation of H_2_O_2_ application, transcriptome sequencing was performed on pea primordial roots under different treatments for 72 h. Eukaryotic reference transcriptome (RNA-seq) analyses of nine samples with PCA (Figure S4A) showed high similarity among the biological replicates. FC ≥ 1.5 and FDR < 0.05 were used as screening criteria in the comparison groups of CK vs. CK1, CK vs. T5, and CK1 vs. T5. In these analyses, 2701 differentially expressed genes (1200 up-regulated and 1501 down-regulated), 6857 differentially expressed genes (3242 up-regulated and 3615 down-regulated), and 6683 differentially expressed genes (3139 up-regulated and 3544 down-regulated) were found, respectively (Figure S4B). The Venn diagram of all differential genes (Figure S4C) showed that the total number of differentially expressed genes in the three control groups was 16,848. In addition, there were 456, 630, and 187 specifically expressed differential genes in the CK vs. CK1, CK vs. T5, and CK1 vs. T5 comparison groups, respectively. Nine DEGs were selected based on this transcriptome analysis (Figure 2), including two IAA-related genes (*GAT1*, *IAA26*), two ABA-related genes *PYL4*, the GA-related gene *PAT1*, the IAA-binding gene *ABP19A* in the cell wall, one lignin synthesis-related gene *CCR1*, and three genes related to starch and sucrose metabolism (*PGM1*, *SUS*, and *BAM3*). These RNA-Seq FPKM values of the genes showed a similar trend to the relative expression by qRT-PCR, confirming the authenticity of the transcriptome data.

### 2.3. Metabolomic Analysis of Pea Primary Roots under H_2_O_2_ and CaCl_2_ Treatments

To further elucidate the mechanism of root growth under the effect of H_2_O_2_ alleviated by CaCl_2_, we performed qualitative and quantitative metabolomic analyses on nine samples, and a total of 647 metabolites were detected. In the CK vs. CK1, CK vs. T5, and CK1 vs. T5 comparison groups, 303 differential metabolites (176 up-regulated and 127 down-regulated), 257 differential metabolites (81 up-regulated and 176 down-regulated), and 280 differential metabolites (124 up-regulated and 156 down-regulated) were found, respectively (Figure S4D). Correlation analysis showed that the correlation coefficients (R^2^) of the biological replicates of the samples were all greater than 0.9 (Figure S4E), and the PCA showed that the samples from different treatments were better separated (Figure S4F), suggesting that the metabolomic data were highly credible.

### 2.4. GO Enrichment Analysis of DEGs

The enrichment of DEGs in GO classification was analyzed at a threshold of *p* < 0.05 for the CK vs. CK1 and CK1 vs. T5 comparison groups, with 2133 and 5132 DEGs, respectively (Table S5). The results showed that the DEGs from both comparison groups were co-enriched in biological processes such as the “response to oxidative stress (GO:0006979)”, “plant-type cell wall organization (GO:0009664)”, and the “hydrogen peroxide catabolic process (GO:004274)”, and enriched in such cellular components as “plant-type cell wall (GO:0009505)”, “cell wall (GO:0005618)”, “integral component of membrane (GO:0016021)”, etc. The molecular functions were enriched in “heme binding (GO:0020037)”, “peroxidase activity (GO:0004601)”, “DNA-binding transcription factor activity (GO:0020039)”, etc. (Figure S5). The biological processes specifically enriched for CK vs. CK1 were the “defense response (GO:0006952)”, “abscisic acid-activated signaling pathway (GO:0009738)”, and “glutamine metabolic process (GO:0006541)”.

The results of the GO enrichment analysis indicated that the alteration of pea primary root growth by exogenous CaCl_2_ and H_2_O_2_ may be achieved through oxidative stress, the alteration of cell wall components, and the activation of phytohormone signaling.

### 2.5. CaCl_2_ and H_2_O_2_ Affect Oxidative Stress within Pea Primary Roots

**Figure 3:**
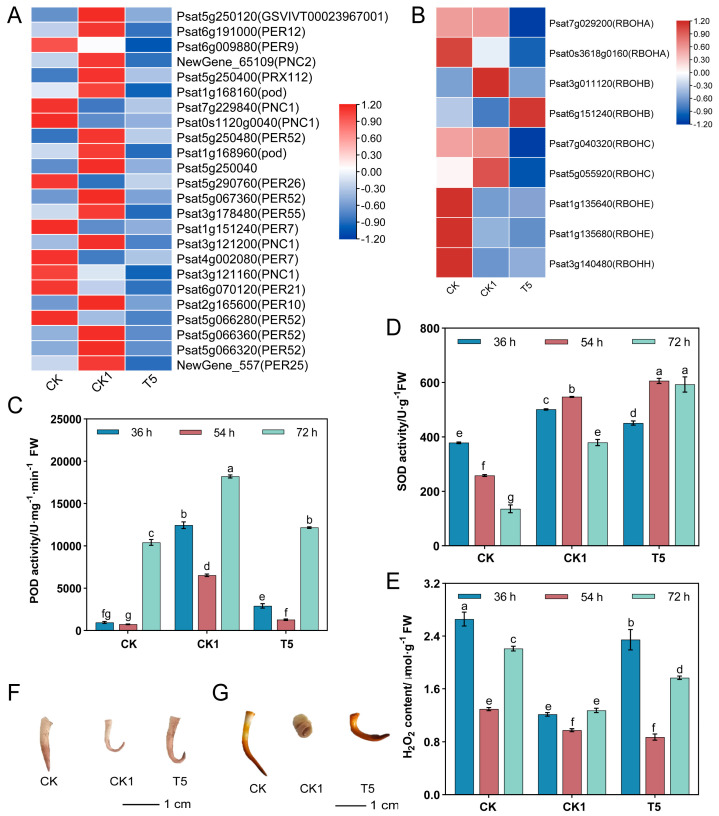
Oxidative stress regulates pea RSA growth. (**A**) Heat map of peroxidase gene expression in the “phenylpropane metabolic pathway”. (**B**) Heat map of respiratory burst oxidase (RBOH) gene expression. (**C**) POD activity. (**D**) SOD activity. (**E**) Endogenous H_2_O_2_ content. Different lower-case letters are the results of significance analyses of Duncan’s multiple range test, indicating statistically significant differences (*p* < 0.05). What is compared here is the significance between different treatments at all times. (**F**) H_2_O_2_ staining of primary roots, wherein pea primary roots cultured for 72 h were stained in DAB staining solution for 2 h. Photographs were taken to observe the staining results. (**G**) Primary root starch staining, wherein pea primary roots cultured for 72 h were stained in Lugol’s iodine solution for 10 min, and photographs were taken to observe the staining results.

The “hydrogen peroxide catabolic process (GO:004274)” and “response to oxidative stress (GO:0006979)”, which were significantly enriched in the GO biological processes, were analyzed for KEGG enrichment to further refine the DEGs-enriched pathways. It was found that the above DEGs were heavily enriched in the “phenylpropanoid biosynthesis” (ko00940) pathway (Figure S6A,B). A total of 24 DEGs were expressed in the “phenylpropanoid biosynthesis” pathway in the CK vs. CK1 and CK1 vs. T5 comparative groups, which were mainly the key genes regulating peroxidase. The heat-map visualization of DEGs co-expressed in the CK vs. CK1 and CK1 vs. T5 comparison groups (Figure 3A) revealed that 9 were up-regulated and 15 were down-regulated in the CK group, 15 were up-regulated and 9 were down-regulated in the CK1 group, and all of them were down-regulated in the T5 group, compared with the 3 treatment groups. By determining the POD (Figure 3C) and SOD (Figure 3D) activities of pea primary roots at 36-, 54-, and 72-h time intervals, it was found that the trends of the enzyme activities were basically the same at the different time intervals. Among them, the SOD and POD activities of the CK1 group were significantly higher than those of the CK group after 72 h. Compared with the CK1 group, the SOD activity of the T5 group was elevated, while the POD activity was significantly lower.

Oxidative stress in the root system alters the endogenous ROS content. DEG enrichment showed (Figure 3B) that the respiratory burst oxidase gene (*RBOH*) was activated by CaCl_2_ and H_2_O_2_ treatment, and the endogenous H_2_O_2_ content of the root system was significantly reduced by exogenous H_2_O_2_ treatment (Figure 3E). In contrast, the endogenous H_2_O_2_ content after the application of exogenous CaCl_2_ on the basis of H_2_O_2_ stress increased significantly compared with CK1, but was lower than that of the CK treatment, and the trends of the H_2_O_2_ content were basically the same at different time intervals. After the DAB staining of pea primary roots cultured for 72 h in the CK, CK1, and T5 treatment groups, the results showed (Figure 3F) that the primary roots were more lightly colored than the CK group under the action of exogenous H_2_O_2_, whereas the CaCl_2_ alleviation treatment resulted in deeper primary root coloration than the H_2_O_2_ treatment. This further indicated that CaCl_2_ might alleviate the inhibition of exogenous H_2_O_2_ on pea primary root growth by regulating endogenous H_2_O_2_.

### 2.6. Effect of CaCl_2_ and H_2_O_2_ on the Contents of Starch and Soluble Sugar

**Figure 4:**
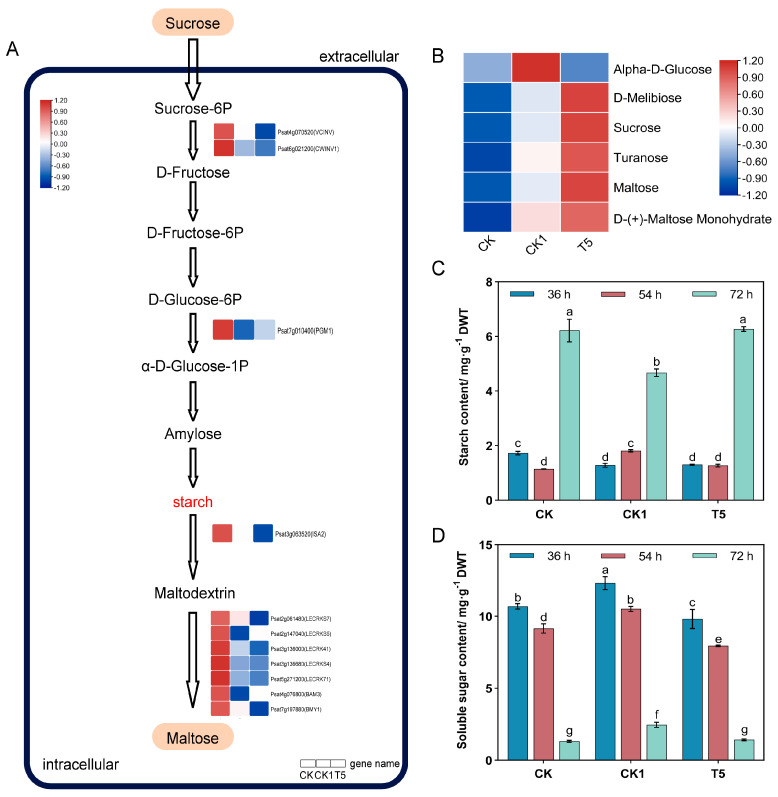
Starch metabolism regulates pea RSA growth. (**A**) The transcriptome and metabolome were combined to analyze the “starch and sucrose metabolic pathway”. (**B**) Heat map of the relative sugar contents as detected by the metabolome. (**C**) Starch content. (**D**) Soluble sugar content. Different lower-case letters are the results of significance analyses of Duncan’s multiple range test, indicating statistically significant differences (*p* < 0.05). What is compared here is the significance between different treatments at all times. Red and blue boxes indicate up- and down-regulated genes, respectively. The main stem of the graph represents the key metabolites obtained based on the KEGG database (DAMs detected by the metabolome have an orange background). The normalized mean expression of each gene is represented by a colored cell, based on a color scale.

The transcriptome and metabolome analyses revealed that some DEGs and DAMs were enriched in the “starch and sucrose metabolic pathway”. Further analysis of this metabolic pathway showed that the expression of 11 DEGs (*VCINV*, *ISA2*, *PGM1*, *ISA2*, *LECRKS7*, *LECRKS5*, *LECRKS4*, *LECRK71*, *BAM3*, and *BMY1*) was significantly down-regulated by exogenous H_2_O_2_ (Figure 4A). After alleviation by CaCl_2_, the expression of three DEGs (*ISA2*, *LECRKS7*, and *BMY1*) was significantly down-regulated and *BAM3* was significantly up-regulated. This suggests that CaCl_2_ and H_2_O_2_ may induce starch synthesis and metabolism in the primary roots of pea plants by regulating the expression of genes related to the “starch and sucrose metabolic pathway”.

Five key soluble sugars were detected in the metabolome (Figure 4B), and the results showed that “D-Melibiose, Sucrose, Turanose, D-(+)-Maltose Monohydrate, and Maltose” abundance was up-regulated in response to H_2_O_2_ and up-regulated after CaCl_2_ alleviation compared to the CK1 group. By determining the contents of starch (Figure 4C) and soluble sugar (Figure 4D) in the primary roots at different time intervals, it was found that the differences in the starch and soluble sugar contents at 36 h and 54 h were smaller. In contrast, the soluble sugar content in pea primary roots significantly increased by 1.87-fold, while starch content significantly decreased under the effect of H_2_O_2_ at 72 h. The soluble sugar and starch contents in the T5 group almost recovered to the level of the CK group. It suggests that it is possible for CaCl_2_ administration to alleviate the inhibition of root gravitropism by H_2_O_2_ by regulating the content of starch.

To investigate the effects of exogenous H_2_O_2_ and CaCl_2_ treatments on the amount and distribution of starch accumulation, we stained pea primary roots with Lugol’s iodine solution. The results showed (Figure 3G) that the starch in the CK group was mainly distributed in the root tip and the coloring was darker; in the CK1 group, the starch was uniformly distributed throughout the primordial roots and the coloring became lighter than that of the CK group; in the T5 group, the starch was distributed throughout the primordial roots and the coloring became darker than that of the CK1 group. Overall observation, followed by the freehand sectioning of transverse sections of primordial roots (Figure S7), revealed that the starch granules were significantly enlarged after H_2_O_2_ treatment, and the size of the starch granules was restored to the CK level after CaCl_2_ alleviation.

### 2.7. Effect of CaCl_2_ and H_2_O_2_ on Calcium Signaling in Pea Primary Roots

**Figure 5:**
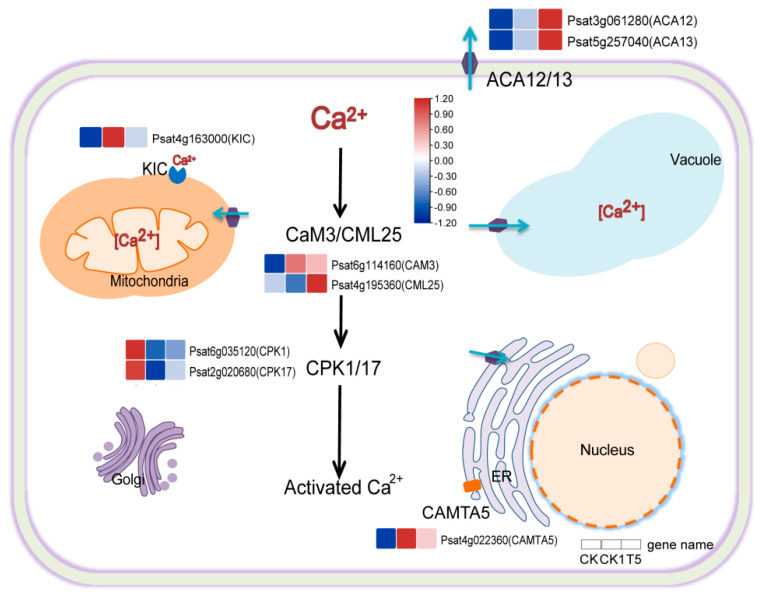
Calcium signaling is involved in pea RSA growth. Red and blue boxes indicate up- and down-regulated genes, respectively. Black arrows: the putative regulatory relationships of key genes for calcium signaling (calcium in the cytoplasm in the presence of exogenous H_2_O_2_ may bind to calmodulin proteins such as CaM, CML25, and CPK1/17, thereby activating calcium signaling). The normalized mean expression of each gene is represented by a color-scale-based colored cell. The ICONS on organelles and cell membranes represent calcium transporters. The purple hexagon (ACA12/13) and orange rectangle (CAMTA5) are calcium channel proteins, and the blue icon is calcium ion binding protein (KIC).

The starch in pea primary roots responds to gravity by activating the calcium signaling pathways. The transcriptome results showed that some DEGs are associated with calcium signaling. Calcium-transporting ATPase (*ACA12/13*), calcium-dependent protein kinase (*CPK1/17*), calcium-binding protein (*KIC*), calmodulin-3 (*CAM3*), and probable calcium-binding protein (*CML25*) with calmodulin-binding transcription activator 5 (*CAMTA5*) were activated under H_2_O_2_ and CaCl_2_ treatment (Figure 5). The predicted subcellular localization of the corresponding proteins of the above eight DEGs by the (https://wolfpsort.hgc.jp/, accessed on 6 November 2023) website revealed that these proteins were mainly distributed on the cytoplasmic membrane (*ACA12/13*), cytoplasm (*CPK1/17*, *CAM3*, and *CML25*), mitochondrion (*KIC*), and endoplasmic reticulum (*CAMTA5*).

### 2.8. Effects of CaCl_2_ and H_2_O_2_ on Phytohormone Signal Transduction in Pea Primary Roots

Transcriptome analysis showed that a large number of DEGs were enriched in “Plant hormone signal transduction (ko04075)”, and the key genes regulating phytohormone signaling under different treatments will be analyzed in the following.

**Figure 6:**
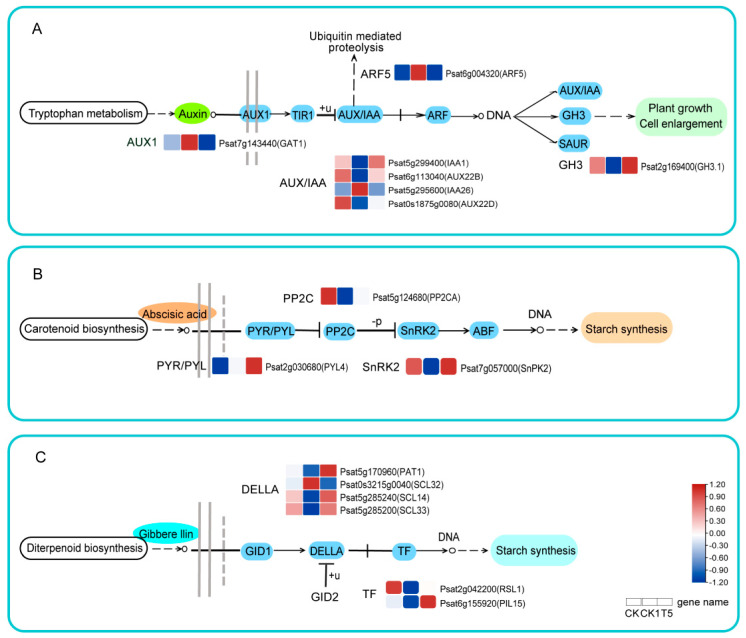
Key genes regulating IAA, ABA, and GA signaling. (**A**) IAA signaling. (**B**) ABA signaling. (**C**) GA signaling. Red and blue boxes indicate up- and down-regulated genes, respectively. The trunk of the graph represents key genes obtained based on the KEGG database. The normalized mean expression of each gene is represented by a color cell based on the color scale. All blue backgrounds in the figure represent key protein regulatory pathways of signaling pathways.

A total of 35 DEGs were detected in the IAA signaling pathway, and 7 DEGs were co-expressed in the CK vs. CK1 and CK1 vs. T5 comparison groups (Table S6). GABA transporter 1 (*GAT1*), auxin-responsive protein IAA26 (*IAA26*), and auxin response factor 5 (*ARF5*) were up-regulated by 2.668, 2.008, and 2.375-fold, respectively, under H_2_O_2_ treatment, and down-regulated by 11.756, 2.040, and 2.438-fold, respectively, in the treatment group of applied CaCl_2_. Probable auxin-responsive protein 1 (*IAA1*) and indole-3-acetic acid-amido synthetase (*GH3.1*) were down-regulated by 2.303 and 2.058-fold, respectively, upon H_2_O_2_ treatment, and CaCl_2_ imposition alleviated the expression of the down-regulated genes (Figure 6A).

A total of 18 DEGs were detected in the ABA signaling pathway, and 3 DEGs were co-expressed in the CK vs. CK1 versus CK1 vs. T5 comparison group (Table S6). Among them (Figure 6B), the abscisic acid receptor (*PYL4*) was up-regulated 1.874- and 2.818-fold in CK vs. CK1 and CK1 vs. T5, respectively. In addition, protein phosphatase 2C (*PP2CA*) was down-regulated 1.794-fold and 1.297-fold in CK vs. CK1 and CK1 vs. T5, respectively, while serine/threonine-protein kinase (*SAPK2*) was down-regulated by 2.236-fold in CK vs. CK1 and up-regulated by 2.359-fold in CK1 vs. T5.

A total of 31 DEGs were detected in the GA signaling pathway, and 6 DEGs were co-expressed in the CK vs. CK1 and CK1 vs. T5 comparison groups (Table S6). Among them, the scarecrow-like transcription factor (*PAT1*), scarecrow-like protein (*SCL14/33*), and transcription factor phytochrome-interacting factor-like 15 (*PIL15*) were down-regulated by 2.330, 1.417, 1.546, and 1.476-fold, respectively, in the CK vs. CK1 comparison group, and were up-regulated by 4.103, 1.591, 1.616, and 2.082-fold, respectively, in the CK1 vs. T5 comparison group (Figure 6C). This shows that pea primordial roots with applied CaCl_2_ under exogenous H_2_O_2_ treatment significantly regulated the expression of key genes in phytohormone signaling.

### 2.9. CaCl_2_ and H_2_O_2_ Affect Pea Primary Root Cell Walls

**Figure 7:**
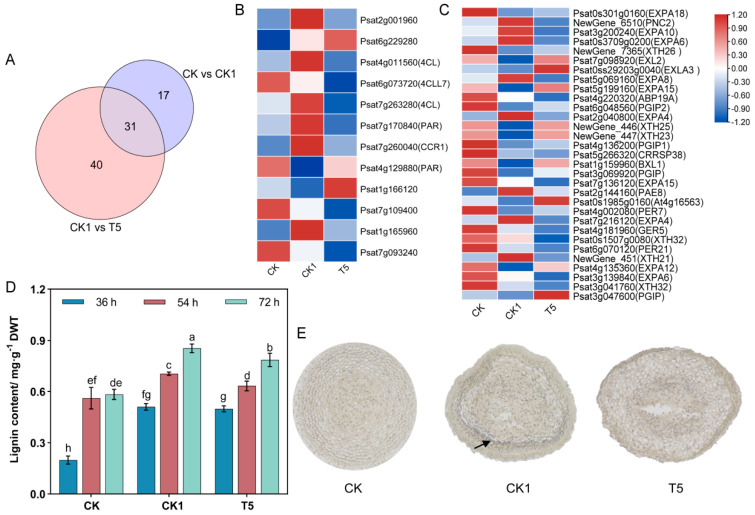
Cell wall involvement in pea RSA growth. (**A**) Venn diagram of the DEGs associated with the “plant cell wall synthesis” pathway. (**B**) Heat map of the expression of the key genes for lignin synthesis. (**C**) Heat map of the DEGs regulating the cell wall relaxation factors. (**D**) Lignin content. Different lower-case letters are the results of significance analyses of Duncan’s multiple range test, indicating statistically significant differences (*p* < 0.05). What is compared here is the significance between different treatments at all times. (**E**) Resorcinol lignin staining. Sections of 72-h pea primary roots were taken and stained with resorcinol, then quickly placed under a light microscope (×100) to observe the degree of lignin accumulation. The darker the color, the greater the lignin accumulation. The black arrows point to layers of cells where lignin accumulates more.

In the “plant-type cell wall (GO:0009505)” and “cell wall (GO:0005618)” pathways, the CK vs. CK1 and CK1 vs. T5 groups were significantly enriched in 48 and 71 DEGs, respectively (Figure 7A), with a total of 31 genes expressed. Exogenous H_2_O_2_ and CaCl_2_ activated the cell wall relaxation factors: α-expansin (*EXPA*) and xyloglucan endotransglucosylase/hydrolase (*XTH*). A heat-map visualization of the key genes showed (Figure 7C) that *EXPA18/10/6/3/8/15/4/12* and *XTH26/25/23/32/21* were differentially expressed in the CK vs. CK1 and CK1 vs. T5 treatment groups. In the CK vs. CK1 group, *EXPA10/6/8/4* and *XTH21* were up-regulated and *EXPA18/12/15/6* and *XTH26/25/23/32* were down-regulated; in the CK1 vs. T5 group, *EXPA3/15/2/12* and *XTH23/25* were up-regulated and *EXPA10/6/8/4* and *XTH21* were down-regulated.

Exogenous H_2_O_2_ and CaCl_2_ treatments induced the differential expression of key genes for lignin synthesis in pea primary roots. A total of 37 DEGs were detected in the lignin synthesis pathway in phenylpropane metabolism, and 12 DEGs were co-expressed in the CK vs. CK1 and CK1 vs. T5 comparison groups (Figure 7B). Among them, phenylalanine ammonia-lyase class 3 (Psat2g001960), 4-coumarate--CoA ligase (Psat4g011560) (Psat7g263280), cinnamoyl-CoA reductase 1 (*CCR1*), phenylacetaldehyde reductase (Psat7g170840), and berberine bridge enzyme-like 21 (Psat1g165960) were up-regulated 2.064-, 2.074-, 2.118-, 6.071-, 2.118-, and 2.809-fold under H_2_O_2_ treatment and down-regulated 1.975-, 2.861-, 3.630-, 3.688-, 3.630-, and 2.143-fold after CaCl_2_ mitigation, respectively. By determining the lignin content of primary roots in the different treatment groups in different time periods, it was found that lignin significantly accumulated under the effect of H_2_O_2_ (Figure 7D), and the lignin accumulation increased with time. To investigate the effects of exogenous H_2_O_2_ and CaCl_2_ treatments on the amount and distribution of lignin accumulation, we stained pea primordial roots with resorcinol. The results showed (Figure 7E) that the CK group had lighter coloring and less lignin accumulation; the coloring of primary roots in the CK1 and T5 groups was deeper than that in the CK group. By determining the lignin content of the primordial roots in different treatment groups, it was found that lignin significantly accumulated under the action of H_2_O_2_, and its content was reduced after CaCl_2_ alleviation (Figure 7E).

## 3. Discussion

The growth morphology of pea primary roots was altered under the action of exogenous H_2_O_2_, while the application of CaCl_2_ could alleviate the phenomenon [25]. In order to investigate the intrinsic mechanism, this study was carried out by applying different concentrations of H_2_O_2_ and CaCl_2_, counting the relevant growth indexes (germination potential, bending rate, and bending degree), and determining the relevant physiological indexes (POD, SOD, lignin content, etc.). Through transcriptome sequencing and metabolome assays, we analyzed the key pathways of DEG and DAM enrichment in pea primary roots under different treatments, with a view to finding the key genes and metabolite interactions of H_2_O_2_ and CaCl_2_ that regulate root growth and development.

### 3.1. CaCl_2_ and H_2_O_2_ Treatments Affect Pea Primary Root Growth toward Gravitropism

The growth and development of the primary root is essential for the early growth of peas [26]. In this study, the germination potential of pea seeds increased in the presence of high concentrations of H_2_O_2_ (Table S7 and Figure S8), suggesting that H_2_O_2_ could promote the germination rate of pea seeds, thereby verifying the findings of Barba-Espin et al. [27]. Jiang et al. [28] found that H_2_O_2_ caused the non-directional growth of the primary roots of *Lathyrus quinquenervius* and wavy growth of the primary roots of *Arabidopsis*, which may be caused by the uneven distribution of calcium ions and IAA [29]. In our study, we found that the geotropism-related growth of pea primordial roots was inhibited under H_2_O_2_ treatment, while the bending rate and bending degree of pea primordial roots increased as the concentration of exogenous H_2_O_2_ increased (Figure 1). This inhibition was relieved by the application of CaCl_2_, which is consistent with the findings of Li et al. [25]. To further investigate whether the alleviation was caused by Ca^2+^ or Cl^−^, this study demonstrated that Ca^2+^ was a key factor in alleviating the inhibition of pea gravitropism by H_2_O_2_ by replacing the counterion to apply CaSO_4_ and KCl with the same particle concentration (Ca^2+^, Cl^−^) as CaCl_2_. However, the exact manner of its mitigation is currently unknown. Root tips usually have more gravity-sensing signals; however, it has been found that plants can still sense gravity after the root crown has been removed [30,31], suggesting that the root tip is not the only gravity-sensing site [32]. The gravity-oriented nature of the roots was weakened after the removal of the medial columella cells from the roots of maize seedlings, but the tendency of gravity-oriented growth was maintained [33]. Therefore, the whole primary root was selected for analysis in this study, in order to screen the key factors of exogenous H_2_O_2_ and the CaCl_2_ regulation of root growth more comprehensively.

### 3.2. Activation of Oxidative Stress in Primary Roots by CaCl_2_ and H_2_O_2_ Treatments

ROS play an important role in shaping the RSA by regulating root growth and lateral root formation [34]. Transcriptome analysis showed that the applied H_2_O_2_ induced oxidative stress in the primary roots, and the enriched pathway was the “phenylpropane metabolic pathway”. The DEGs in this pathway mainly regulate peroxidase. The study by Wan et al. [35] showed that exogenous H_2_O_2_ treatment could enhance the cold resistance of oilseed rape seedlings by inducing the accumulation of antioxidant substances and activating the activity of antioxidant enzymes. In this study, we found that 11 peroxidase genes (including *GSVIVT00023967001*, *PER12*, *PNC2*, *PRX112*, *POD*, *PER52*, *Psat5g250040*, *PER55*, *PNC1*, *PER10*, and *PER25*) were up-regulated in response to H_2_O_2_, whereas all of them were down-regulated under CaCl_2_ treatment. Barley root growth under high Cd stress was inhibited, while cationic POD isozymes accumulated Cd and were concentration-dependent [36]. In this study, the POD and SOD activities in primary roots were significantly elevated by H_2_O_2_, while POD activity was reduced by CaCl_2_ alleviation. SOD is at the core of antioxidant enzymes, and its elevated activity scavenges free radicals and enhances membrane permeability [37]. It was found that exogenous Ca^2+^ could improve the antioxidant capacity of black algae to enhance its resistance to Cd [38].

ROS have a dual role in the plant body, one as stressors that trigger oxidative stress and the other as signaling molecules that are involved in plant development [39]. It was found that respiratory burst oxidase D (RBOHD) induces the production of cytoplasmic ectodomain ROS [40]. In this study, exogenous H_2_O_2_ stress led to a decrease in endogenous H_2_O_2_ content (Figure 3E), whereas the content of endogenous H_2_O_2_ in the root system increased after CaCl_2_ application. This suggests that Ca^2+^ may be regulating the endogenous H_2_O_2_ to alleviate the non-gravitropic nature of primary roots due to exogenous H_2_O_2_ stress, which is similar to the findings of Liu et al. [41]. It was found that endogenous H_2_O_2_ synthesis was reduced by the exogenous H_2_O_2_ inhibition of sallow bean [28] root vigor, while the endogenous H_2_O_2_ content of pea primordial roots [41] was significantly reduced by the activation of antioxidant enzyme systems. We further confirmed that exogenous H_2_O_2_ inhibited endogenous H_2_O_2_ accumulation using DAB staining (Figure 3F), and hypothesized that this was possibly because exogenous H_2_O_2_ accelerated the clearance of endogenous H_2_O_2_ by POD; the specific mechanism of action needs to be further explored. Transcriptome analysis showed that the expression of RBOH-related genes (*RBOHA*, *RBOHB*, *RBOHC*, *RBOHE*, and *RBOHH*) was activated upon the application of H_2_O_2_. This suggests that *RBOH* may be a key gene in the exogenous H_2_O_2_ regulation of endogenous H_2_O_2_ content changes. It was found that the expression of *AtRBOHC* regulates root development in *Arabidopsis* [42], and it can be hypothesized that the *RBOH* gene in this study may be related to pea root development, which needs further verification.

### 3.3. Effect of CaCl_2_ and H_2_O_2_ on Starch Metabolism in Primary Roots

According to the starch-equilibrium body hypothesis, starch-filled amyloplasts are asymmetrically distributed in the root system during gravity perception, thus inducing asymmetric growth signal transmissions [43,44]. In this study, exogenous H_2_O_2_ decreased the starch content and increased the soluble sugar content in primary roots. Exogenous H_2_O_2_ also decreased the distribution of starch in the root tip, which finding is similar to the findings of Zhou et al. [29]. It suggests that H_2_O_2_ may attenuate root gravitropism by converting starch to sugar in the root tip. The application of CaCl_2_ could also alleviate the acceleration of starch metabolism induced by H_2_O_2_, thus restoring the gravity-oriented nature of roots to some extent. Interestingly, the starch granules were significantly enlarged under the H_2_O_2_ treatment compared to the CK treatment, which may also be a key factor leading to the change in gravitropism, the exact mechanism of which remains to be further verified.

Starch consists of straight-chain starch and branched-chain starch, in which straight-chain starch is synthesized by granule-bound starch synthase (*gss1*) activity [45,46]. It was found that the reduced expression of the *PGM1* gene, a key starch-synthesizing gene in the root tip of *Arabidopsis*, resulted in diminished root geotropism [47]. In contrast, in *pgm1* mutants, the deposition of amyloid-free plastids is blocked, leading to a slowing of the gravitropic response in roots and shoots [48]. In this study, the transcriptome and fluorescence quantification results showed that H_2_O_2_ down-regulated the expression of *PGM1* (Figure 2 and Figure 4A), a key gene for starch synthesis, and its expression was up-regulated after CaCl_2_ alleviation. It has been found that Ca^2+^ stabilizes α-amylase activity modulating gravitational sensitivity; therefore, Ca^2+^ and amylase are the controlling factors in stabilizing starch content in cells [49,50,51]. Combined with the results of starch content measurements, we can speculate that *PGM1* is a key gene in the H_2_O_2_-regulated changes seen in starch content in pea primary roots.

### 3.4. Effects of CaCl_2_ and H_2_O_2_ on Calcium Signaling in Primary Roots

Instantaneous changes in Ca^2+^ are early events in the plant’s response to a variety of environmental signals [52]. It was found that cold stress induces Ca^2+^ signaling in plant cells, involving the activation of Ca^2+^ channels and Ca^2+^ pumps [53,54]. Water stress causes hypoxia in plant roots, and by knocking down *CAX* (Ca^2+^/H^+^ exchanger) and *ACA* (Ca^2+^-ATPase), it was found that the harmful effects of water stress on roots were alleviated by *ACA* knockdown [55]. In plant cells, CaM, calmodulin neurophosphatase b-like proteins (CBLs), CMLs, and CDPKs (CPKs) can bind to free calcium in the cytoplasm, triggering a conformational change of the proteins that can lead to downstream physiological and biochemical responses [56,57,58]. Ca^2+^ is involved in plant root geotropism. An earlier study found that gravity leads to the asymmetric distribution of Ca^2+^ gradients within pea and maize roots [59], and that the application of Ca^2+^ chelating agents resulted in the retardation of root geotropism [60]. Gravity-stimulated Ca^2+^ is involved in regulating differential changes in extracellular pH in the elongation zones of *Arabidopsis* roots on both the ground-oriented and far-ground sides in response to auxin, resulting in a change in root orientation [61]. In addition, primary *Arabidopsis* roots were less gravity-oriented in the presence of exogenous H_2_O_2_. The expression of *MCA1*, which encodes a Ca^2+^-permeable mechanosensitive channel, was significantly increased and Ca^2+^ levels were higher in cells on the inner side of bent roots than in those on the outer side [29]. Through transcriptome analysis, we predicted the subcellular localization of key genes regulating calcium signaling and found that *ACA12/13*, *CPK1/17*, *KIC*, *CAM3*, *CML25*, and *CAMTA5* were activated by H_2_O_2_ and CaCl_2_. It was further demonstrated that root geotropism was regulated by Ca^2+^.

### 3.5. Effects of CaCl_2_ and H_2_O_2_ on Phytohormone Signal Transduction

The phytohormones IAA, ABA, and GA, which are key regulators of cell elongation and division [62], are essential in the physiology of plant resistance to adversity [20]. The RSA is affected by the crosstalk of different hormones, and external signaling molecules alter the plant signaling pathways [63,64]. In *Arabidopsis*, auxin early and fast-response genes, including auxin/indole-3-acetic acid (*Aux/IAA*), *ARF*, *SAUR*, and *GH3*, are key genes for auxin signaling [65,66]. The auxin-induced *GH3* gene can mediate IAA inactivation through coupling, which, in turn, attenuates auxin signaling [67]. *MP/AtARF5* can control embryonic root initiation by interacting with transcription factors [68]. *AtARF10/16* binds to microRNA160, which regulates root-cap cell formation [69]. In the ABA signaling pathway, protein phosphatase 2Cs (*PP2Cs*) are negative regulators, *PYR/PYL/RCARs* are ABA receptors, and snf1-related protein kinase 2s (*SnRKs*) are positive regulators, which collectively mediate the stimulatory response of the plant body to exogenous signaling molecules [70,71]. In this study, during the action of H_2_O_2_ with CaCl_2_, the hormones regulating IAA (*GAT1*, *IAA26*, *ARF5*, *IAA1*, *AUX22B*, *AUX22D*, and *GH3.1*), ABA (*PYL4*, *PP2CA*, and *SAPK2*), and GA (*SCL32*, *PAT1*, *RSL1*, *SCL14*, *PIL15*, and *SCL33*) key genes for signaling were activated. This may be the reason for the differential growth of pea primary roots induced by H_2_O_2_ and CaCl_2_.

### 3.6. Effect of CaCl_2_ and H_2_O_2_ on the Cell Wall of Primary Roots

Under water stress, the genes regulating those enzymes related to maize isoflavone biosynthesis are up-regulated and lignin is increased in the elongation zone [72]. The phenylpropane pathway is one of the sources of the lignin found in plant cells. POD is the last enzyme in the lignin synthesis pathway and high POD activity increases lignification [73]. Large accumulations of lignin under drought stress limit cell-wall extension in soybean roots [74]. The EXPA and XET proteins play important roles in cell wall expansion [75]. In our study, the genes regulating EXPA and XET showed 20 DEGs up-regulated and 11 DEGs down-regulated in the CK treatment group, 8 DEGs up-regulated and 23 DEGs down-regulated in the CK1 treatment group, and 9 DEGs up-regulated and 22 DEGs down-regulated in the T5 treatment group. This indicates that the curved growth of pea primary roots under H_2_O_2_ treatment may be related to the activities of EXPA and XET proteins. Cell wall extension depends on the deposition of cell wall components and the modification of cell wall structure to balance rigidity and extensibility. It was shown that the cytoskeletal network, the deposition of cell wall components, Ca^2+^ homeostasis, ROS, ectoplasmic pH changes, and cell-wall-modifying proteins regulate cell wall extension [76,77,78]. To better adapt to the external environment, the plant body must establish the correct cell shape and size [79]. Therefore, it can be hypothesized that exogenous H_2_O_2_ may promote lignin accumulation by increasing POD activity [80], thus altering the cell wall extensibility of pea primary roots and affecting the normal growth of root cells. After the application of CaCl_2_, there were no significant changes in the genes regulating the expression of EXPA and XET proteins compared to the CK1 group, while the lignin content was reduced compared to the CK group.

## 4. Materials and Methods

### 4.1. Plant Materials and Treatment

The Longwan 1 pea was used as the experimental material. Pea seeds of uniform size and full grains were selected; they were first rinsed with running water for 30 min, then sterilized with 75% alcohol for 30 s, and finally rinsed with sterile water 4–5 times. The seeds were placed in petri dishes with two layers of filter paper, then 20 seeds were placed in each petri dish. Finally, 20 mL of culture solution was added and the seeds were incubated in the incubator (Zhejiang Topu Yunnong Technology Co., Ltd.; Zhejiang, China) at a constant temperature of 25 °C for 72 h in the dark.

The culture solution concentration was screened with reference to the method used by Li et al. [25]. H_2_O_2_ concentration was screened with six concentration gradients of 0, 20, 80, 150, 200, and 300 mmol·L^−1^ H_2_O_2_ (Sinopharm Group Chemical reagent Co., Ltd.; Shanghai, China). Each petri dish was considered as one replicate, and three replicates were set up. At the four time points of 24, 36, 54, and 72 h, the germination potential, bending rate, and bending degree of peas in different experimental groups were counted; 150 mmol·L^−1^ was determined to be the optimal inhibitory concentration.

For CaCl_2_ concentration screening, a control group with CK (deionized water), CK1 (150 mmol·L^−1^ H_2_O_2_), CK2 (10 mmol·L^−1^ CaCl_2_ (Tianjin Guangfu Technology Development Co., Ltd.; Tianjin, China)), and a mitigation group (H_2_O_2_ + different concentrations of CaCl_2_) (Table 1) were set up to carry out the experiment. The specific treatment concentrations of CaCl_2_ are given in Table 1. Each petri dish was considered as one replicate, and three replicates were set up. At the four time points of 24, 36, 54, and 72 h, the germination potential, bending rate, and bending degree of peas in the different experimental groups were measured. T5 (150 mmol·L^−1^ H_2_O_2_ + 10 mmol·L^−1^ CaCl_2_) was finally determined as the optimal mitigation concentration. The primary roots of the CK, CK1, and T5 groups treated for 36, 54, and 72 h were taken and stored at −80 °C for subsequent index measurements.

### 4.2. Root Germination Potential, Bending Rate and Bending Degree Statistics

The germination potential, bending rate, and bending degree of pea seeds under different treatments were measured and 3 replications for each were performed (1 replicate per petri dish, 20 seeds per dish).

The statistical criterion for germination potential is as follows [80]: the length of the radicle of the seed is equal to the length of the seed.

Germination potential (%) = (number of seeds germinating normally at 72 h/total number of seeds per dish).

The statistical criterion for bending primary roots is as follows [41]: primary roots were considered to be bent if the angle between the tip growth angle and the direction of gravity was greater than 180°, i.e., bent, and the bending angle was measured using ImageJ 1.53q software (https://imagej.net/, accessed on 17 July 2023) and Java 1.8.0_322 (64-bit) [81].

### 4.3. Measurement of Physiological Indices

Starch content, soluble sugar content, lignin content, superoxide dismutase (SOD) (EC 1.15.1.1) activity, peroxidase (POD) (EC 1.11.1.7) activity, and hydrogen peroxide content were determined using assay kits (Suzhou Keming Biotechnology Co.; Ltd.; Suzhou, China).

### 4.4. H_2_O_2_, Starch, and Lignin Staining

The diaminobenzidine (DAB) method was used to stain the primary roots of pea plants to detect H_2_O_2_ changes [82,83]. Primary roots from CK, CK1, and T5 specimens treated for 72 h were taken and placed in 1 mg·mL^−1^ DAB (Hefei Bomei Biotechnology Co., Ltd.; Hefei, China) staining solution, then treated in the dark at a constant temperature of 25 °C for 2 h. The DAB solution was poured out, and the primordial roots were rinsed with distilled water 4–5 times to remove the staining solution on the surface of the material, and the staining was observed; the darker the yellow color, the greater the H_2_O_2_ content.

The pea primary roots were stained using Lugo’s iodine solution [84] (iodine 4.5–5.5% (Tianjin Guangfu Technology Development Co., Ltd.; Tianjin, China) and potassium iodide 9.5–10.5% (Tianjin Guangfu Technology Development Co., Ltd.; Tianjin, China)) method to detect starch changes. Primary roots from the groups with CK, CK1, and T5 treatment for 72 h were placed in 9-millimeter disposable Petri dishes, to which 20 mL of staining solution was added (the concentration of staining solution could be adjusted according to the starch content of the material), and the staining was photographed and observed after 10 min.

Pea root tips were stained using the phloroglucinol method [85] to detect changes in lignin at 72 h under the CK, CK1, and T5 treatments. Pea root tips of 1 cm were placed in 2 mL centrifuge tubes and fixed for more than 24 h by adding 1.8 mL of FAA 70% fixative. Paraffin sections (apical transverse sections) were completed by Wuhan Xavier Biotechnology Co. (Wuhan, China). A total of 1% phloroglucinol (Yuanye Biotechnology technology company, Shanghai, China) solution (95% ethanol) was added dropwise to the sections and stained for 2 min. Then, 25% HCl was added dropwise for 2 min to develop the color. The samples with completed color development were quickly placed under a microscope (Leica Microsystems, Wetzlar, Germany) to observe the lignin distribution throughout the cross-section of the root tip and photographed.

### 4.5. Transcriptome Sequencing

Three biological replicates of pea primary roots from each of the CK, CK1, and T5 treatment groups were made. RNA sequencing analysis was performed by Biomarker Technologies Co., Ltd. (Beijing, China). Total RNA from the pea primary roots was extracted using the RNA Prep Pure Plant Kit (Tiangen, Beijing, China) according to the instructions provided by the manufacturer, and RNA sequencing analysis was performed using the Hieff NGS Ultima Dual-mode mRNA Library Prep Kit for Illumina (Yeasen Biotechnology (Shanghai) Co., Ltd., Shanghai, China). The libraries were finally sequenced on the Illumina NovaSeq platform. The raw data were further processed using the bioinformatics analysis platform BMKCloud (www.biocloud.net, accessed on 5 October 2023). Reads containing adapters were removed from the raw data, and these clean reads were mapped to the reference genome of Pisum_sativum_v1a (GCA_900700895.2, NCBI).

Genes with a DESeq2 corrected *p*-value < 0.05 and a fold change of ≥1.5 [86] were designated as differentially expressed. Differentially expressed genes (DEGs) were functionally annotated using the GO [87] and KEGG [88] databases.

### 4.6. Quantitative Real-Time PCR

To verify the reliability of the transcriptome sequencing data, nine DEGs were selected from the transcriptome data for qRT-PCR. Three replications were made for each treatment. The qRT-PCR primer sequence information is given in Table S8. qRT-PCR was performed after RNA reverse transcription using a LightCycler^®^96 Real time-PCR machine (Roche, Mannheim, Germany). The reaction system used a total of 20 μL: 10 μL of 2 × Talent qPCR premix, 6.8 μL of RNase-Free ddH_2_O, 2 μL of 75 ng/μL cDNA, and the forward and reverse primers of 10 μmol·L^−1^ were 0.6 μL each. The qRT-PCR reaction program was used as referenced by Lu et al. [86]. The relative expression of nine genes was calculated by the 2^−ΔΔCt^ method, using β-tubulin [89] as the housekeeping gene.

### 4.7. LC–MS/MS Analysis

The same samples were used for the metabolome as for the transcriptome. Metabolome assays was performed with the UPLC-ESI-MS/MS system (UPLC, Waters Acquity I-Class PLUS; MS, Applied Biosystems QTRAP 6500+) (Waters, Milford, MA, America). The total peak area was normalized to the original peak area information for subsequent analysis. The screening criteria were: FC > 1, *p*-value < 0.05, and VIP > 1 [86]. Spearman correlation analysis and principal component analysis (PCA) were used to determine the reproducibility of within-group and quality-control samples.

### 4.8. Statistical Analysis

The experimental data were analyzed for statistical significance using Excel 2010 (Microsoft Corporation, Redmond, WA, America) and IBM SPSS Statistics 26 software (IBM Corporation, Armonk, NY, America). Graphs were generated using TBtools v2.096 (Dr. Chengjie Chen, Guangzhou, China, https://github.com/CJ-Chen/TBtools/releases, accessed on 4 August 2024), GraphPad Prism 9 (Dr. Harvey Motulsky, USA), Adobe Illustrator CC 2018 (Adobe Corporation, SAN Jose, CA, America), and Adobe Photoshop 2021.

## 5. Conclusions

**Figure 8:**
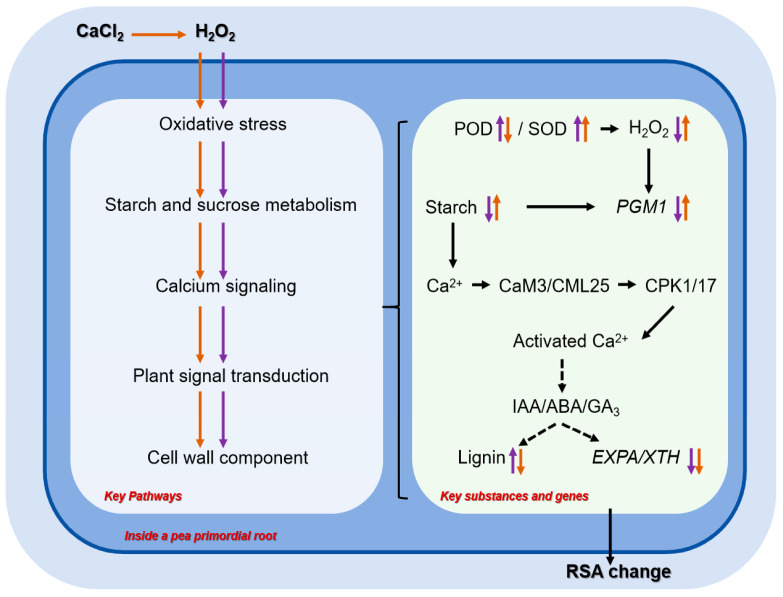
Model of the mechanism by which CaCl_2_ mitigates the H_2_O_2_ inhibition of pea primordial roots toward gravitropism. By summarizing previous research along with the results of the present study, it can be hypothesized that CaCl_2_ may alleviate the mechanism of H_2_O_2_ inhibition of pea primary root growth toward gravitropism. Under the gravitational field, exogenous H_2_O_2_ induced oxidative stress responses in pea primary roots, which mainly consisted of elevated regulatory POD and SOD activities and reduced endogenous H_2_O_2_ content. Meanwhile, the reduced H_2_O_2_ content induced the down-regulation of *PGM1* expression and reduced the starch content. This altered the gene expression pattern of the downstream calcium signaling and phytohormone signaling pathways. We hypothesized that phytohormone signaling may regulate the down-regulation of the cell wall extensin *EXPA/XTH* gene and the increase in lignin accumulation, while the application of CaCl_2_ could alleviate the inhibition of pea primary roots toward gravitropism by exogenous H_2_O_2_. In the present study, CaCl_2_ may have alleviated the inhibition of pea primary root gravitropism by H_2_O_2_ by regulating the level of oxidative stress in primary roots (a decrease in POD activity and an increase in SOD activity), increasing the endogenous H_2_O_2_ and starch content, and decreasing the lignin accumulation to mitigate the inhibition of pea primary root gravitropism by H_2_O_2_. Black text: body content (signaling molecules, signaling pathways, metabolites, etc.) as obtained from the summary of this study. Black arrows: regulatory pathways. Black dashed arrows: possible regulatory pathways. Purple arrows: the regulatory pathways of H_2_O_2_. Red arrows: the regulatory pathway of CaCl_2_.

In this study, we comprehensively revealed the key pathways through transcriptomics and metabolomics that function to alleviate the inhibition of pea primary root gravitropism by H_2_O_2_ with exogenous CaCl_2_ (Figure 8). The transcriptome results indicated that CaCl_2_ may alleviate H_2_O_2_ stress by regulating the pea primary root oxidative stress response, starch and sucrose metabolism, calcium signaling and phytohormone signaling, and cell wall composition under a gravity field. Among them, *PGM1*, which regulates starch synthesis, is a key gene for gravity perception in pea primary roots. The metabolome and physiological and biochemical results showed that CaCl_2_ might alleviate the inhibition of H_2_O_2_ on the gravitropic movement of pea primary roots by regulating POD and SOD activities, increasing the content of endogenous H_2_O_2_ and starch, and decreasing the accumulation of lignin to alleviate the inhibition of H_2_O_2_ on the gravitropic movement of pea primary roots. This study not only contributes to the study of the pathways of pea root growth under adversity stress but also provides theoretical references for the study of root vectorial movement.

## Figures and Tables

**Table 1:** Concentrations of the H_2_O_2_ and CaCl2 treatments.

Treatment	H_2_O_2_ (mmol·L^−1^)	CaCl_2_ (mmol·L^−1^)	Ca^2+^ (mmol·L^−1^)	Cl^−^ (mmol·L^−1^)
CK	0	0	0	0
CK1	150	0	0	0
CK2	0	10	10	20
H1	20	0	0	0
H2	80	0	0	0
H3	200	0	0	0
H4	300	0	0	0
T1	150	1	1	2
T3	150	5	5	10
T5	150	10	10	20
T7	150	15	15	30

Note: Deionized water was used for the configuration of all culture solutions in the experiment, excluding ionic forms other than water molecules.

## Data Availability

To support the results, the RNA-seq raw data set has been deposited in the NCBI database (https://www.ncbi.nlm.nih.gov/bioproject/, accessed on 27 September 2023), with alogin accession of PRJNA1021333.

## Supplementary Materials

The following supporting information can be downloaded at: https://www.mdpi.com/article/10.3390/ijms25168613/s1.
