# Supplementary material for: Intrinsic Mechanism of CaCl2 Alleviation of H2O2 Inhibition of Pea Primary Root Gravitropism
Source: Int J Mol Sci. 2024 Aug 7;25(16):8613. doi: 10.3390/ijms25168613 (PMC11354692; doi:10.3390/ijms25168613)
Supplement: Supplementary file 1 [file ijms-25-08613-s001.zip › Supplementary Materials.pdf]

Intrinsic Mechanism Of $CaCl_2$ Alleviation Of H_2O_2 Inhibition Of Pea Primary Root Gravitropism

Supplementary Data

Table S1 Primary root bending rate of pea under different treatments at different times

Treatments	24 h/%	36 h/%	54 h/%	72 h/%
CK	0±0.00d	0±0.00d	0±0.00f	0±0.00e
CK1	72±0.25a	71±0.04a	50±0.06a	53±0.06a
CK2	0±0.00d	8±0.14d	0±0.00f	0±0.00e
T1	0±0.00d	48±0.13abc	37±0.07abcd	36±0.08be
T3	22±0.19abc	33±0.14c	31±0.06bcde	37±0.06bc
T5	17±0.29cd	42±0.14bc	30±0.11cde	27±0.04cd
T7	17±0.29cd	68±0.03a	42±0.07abc	36±0.02bc
B1	0±0.00d	0±0.00d	0±0.00f	0±0.00e
B2	18±0.17cd	52±0.25abc	44±0.06ab	45±0.05ab
B3	42±0.37abc	41±0.03bc	34±0.04bcd	34±0.05c
B4	33±0.58abc	30±0.12c	21±0.09e	23±0.06d
B5	33±0.58abc	35±0.14c	29±0.06de	33±0.06c
C1	0±0.00d	0±0.00d	0±0.00f	0±0.00e
C2	64±0.34ab	48±0.13abc	42±0.12abc	34±0.05c
C3	64±0.13ab	64±0.19ab	43±0.06ab	47±0.12a
C4	50±0.17abc	49±0.14abc	42±0.07abc	37±0.06bc
C5	28±0.25abc	63±0.10ab	43±0.08abc	33±0.06c

Different lower-case letters are the results of significance analyses of Duncan’s multiple range test, indicating statistically significant differences ($p < 0.05$). The table shows the significance of different processes at the same time.

Table S2 Pea primary root bending degree under different treatments at different times

Treatments	24 h/°	36 h/°	54 h/°	72 h/°
CK	0.000±0.00i	0.000±0.00k	0.000±0.00i	0.000±0.00e
CK1	250.925±2.09a	402.759±4.50b	330.330±2.25c	328.982±6.13b
CK2	0.000±0.00i	0.000±0.00k	0.000±0.00i	0.000±0.00e
T1	0.000±0.00i	156.874±1.44j	60.434±7.09h	382.253±110.06a
T3	128.665±2.38e	241.575±2.11f	184.081±7.43f	216.923±10.02c
T5	70.368±12.54g	192.102±8.01h	162.082±2.99g	133.915±5.19d
T7	105.239±0.83f	373.665±10.93d	275.298±8.21e	235.675±9.79c
B1	0.000±0.00i	0.000±0.00k	0.000±0.00i	0.000±0.00e
B2	208.358±5.44b	367.749±5.00d	363.340±13.77a	293.362±9.26b
B3	153.009±12.79d	291.497±10.13e	296.382±11.16d	220.439±13.33c
B4	61.554±0.88h	229.750±6.58g	148.571±9.24g	215.332±5.76c
B5	174.102±4.56c	181.113±4.38i	336.235±8.63c	290.411±5.57b
C1	0.00±0.00i	0.00±0.00k	0.000±0.00i	0.000±0.00e
C2	243.135±3.41a	388.867±3.40c	337.829±17.61c	222.025±36.57c
C3	214.693±6.57b	378.503±2.65cd	405.667±13.78a	312.252±7.72b
C4	247.032±8.79a	451.004±15.66a	310.974±27.91d	325.068±8.69b
C5	246.394±5.37a	409.964±2.53b	303.909±9.79d	335.912±4.67ab

Different lower-case letters are the results of significance analyses of Duncan's multiple range test, indicating statistically significant differences ($p < 0.05$). The table shows the significance of different processes at the same time.

Table S3 Bending rate and curvature of pea primary roots under $CaCl_2$ mitigated H_2O_2 treatment

Treatments	Bending degree/°	Bending rate/%
CK	0.000±0.00i	0±0.00g
CK1	328.982±6.13c	57±0.06ab
CK2	0.000±0.00i	0±0.00g
H1	197.321±7.09g	18±0.02f
H2	301.077±4.80d	48±0.05c
H3	371.165±4.78b	53±0.04bc
H4	468.997±15.36a	62±0.04a
T1	331.653±22.54c	36±0.08d
T3	216.923±10.01f	37±0.06d
T5	133.915±5.19h	27±0.04e
T7	235.675±9.79e	36±0.02d

Different lower-case letters are the results of significance analyses of Duncan's multiple range test, indicating statistically significant differences ($p < 0.05$). The table shows the significance of different treatments for the same indicator.

Table S4 Seed treatment conditions and numbers

Treatments	H_2O_2 (mmol·L^{-1})	$CaCl_2$ (mmol·L^{-1})	$CaSO_4$ (mmol·L^{-1})	KCl (mmol·L^{-1})
CK	0	0	0	0
CK1	150	0	0	0
CK2	150	0	0	0
T1	150	1	0	0
T3	150	5	0	0
T5	150	10	0	0
T7	150	15	0	0
B1	150	0	0	0
B2	150	0	1	0
B3	150	0	5	0
B4	150	0	10	0
B5	150	0	15	0
C1	150	0	0	0
C2	150	0	0	2
C3	150	0	0	10
C4	150	0	0	20
C5	150	0	0	30

Table S5 Statistics on the number of annotated DEGs

DEG Set	Total	COG	GO	KEGG	KOG
CK vs CK1	2555	885	2133	1734	1172
CK1 vs T5	6132	2031	5132	4324	3267

Table S6 Phytohormone signal transduction DEGs

Comparison group	IAA	GA	ABA
CK vs CK1	16	8	11
CK1 vs T5	26	13	26
CK vs CK1 vs T5	7	3	6

Table S7 Germination potential of primary roots of pea under different treatments at different times

Treatments	24 h/%	36 h/%	54 h/%	72 h/%
CK	0±0.00f	50±0.10ef	87±0.15a	97±0.06ab
CK1	20±0.10de	70±0.10abcd	93±0.06a	100±0.00a
CK2	0±0.00f	33±0.06g	63±0.15b	93±0.06ab
T1	3±0.06f	57±0.06cedf	90±0.10a	93±0.06ab
T3	27±0.06bcd	47±0.12fg	87±0.12a	100±0.00a
T5	10±0.10ef	73±0.12abc	80±0.10ab	97±0.06ab
T7	10±0.10ef	63±0.06bcde	80±0.00ab	83±0.06c
B1	3±0.06f	67±0.06abcde	90±0.00a	90±0.00bc
B2	40±0.10ab	70±0.20abcd	77±0.15ab	97±0.06ab
B3	37±0.12abc	73±0.06abc	90±0.10a	970.06ab
B4	10±0.00ef	57±0.06cdef	93±0.06a	100±0.00a
B5	10±0.10ef	70±0.10abcd	93±0.06a	100±0.00a
C1	0±0.00f	53±0.06def	80±0.00ab	93±0.06ab
C2	30±0.10abcd	83±0.06a	87±0.06a	97±0.06ab
C3	30±0.10abcd	80±0.10ab	93±0.12a	100±0.00a
C4	43±0.06a	67±0.06abcde	80±0.00ab	100±0.00a
C5	23±0.06cde	73±0.06abc	93±0.06a	100±0.00a

Different lower-case letters are the results of significance analyses of Duncan's multiple range test, indicating statistically significant differences ($p < 0.05$). The table shows the significance of different processes at the same time.

Table S8 Primer sequences

Gene name	Primer sequenes	XM name
GAT 2.1	Forward: CCACATCAATACACCATCACTTGTTC<br>Reverse: AGAATGACGGAGAGATCGGAAGAC	XM_051055846.1
IAA26	Forward: GAGGATAATGAAGGAGACAGGATGC<br>Reverse: TGCTTAGTAGTCCCAAGTGTAAATGC	XM_051028312.1
CCR1	Forward: TGTTGTCGTTGATGAGTCGTATTGG<br>Reverse: TTCCGCCACTGTCTTCCCATAAC	XM_051038198.1
ABP19A	Forward: AGCACGACTAGATATAGCCGAAGG<br>Reverse: ATCCAGCAGTTATTTCTCCTTGAACC	XM_051017879.1
SUS2	Forward: CTTGTAGATGGAACGGATAGTGACTG<br>Reverse: GAAACTGAACACCATTGCCAATAGAC	XM_051029776.1
PYL4	Forward: CGCTGGCTCAGATCGCAGAG<br>Reverse: CCGACCCGAAACCCGTATTGG	XM_051048262.1
PAT1	Forward: GTTGTCAACTTAGTAGCCTGCGAAG<br>Reverse: GTGAATCCAGCCCTTGCGAAAC	XM_051025303.1
PGM1	Forward: TTCCCCTTCGCACAAACATAATCAC<br>Reverse:GAGGTTCCAGGCTTCTGTCCATC	XM_051045647.1
BAM3	Forward: CTCATAACAACTTCTCTGGCTCAATTC<br>Reverse: TGATGAAGAACGGTTACATGGACTC	XM_051047936.1
beta-1	Forward: TGGTACACAGGTGAAGGAATGGATG<br>Reverse: TCCTCATCAGCAGTCGCATCTTG	XM_051045246.1

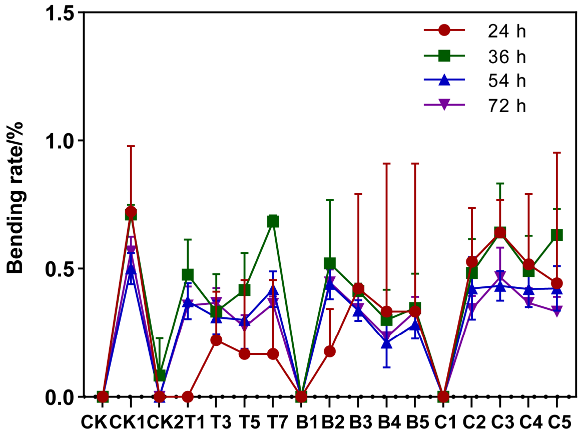

Figure S1 Primary root bending rate of pea under different treatments at different times

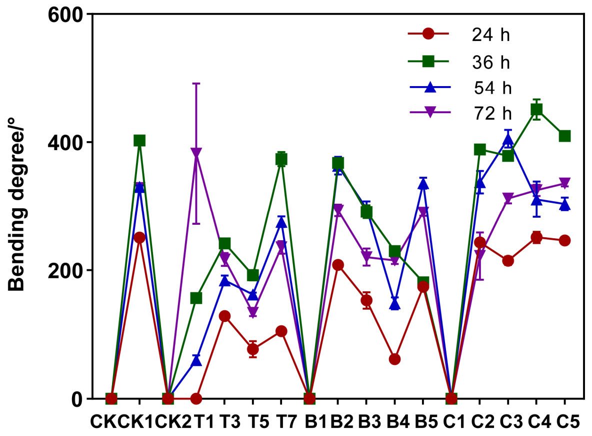

Figure S2 Pea primary root bending degree under different treatments at different times

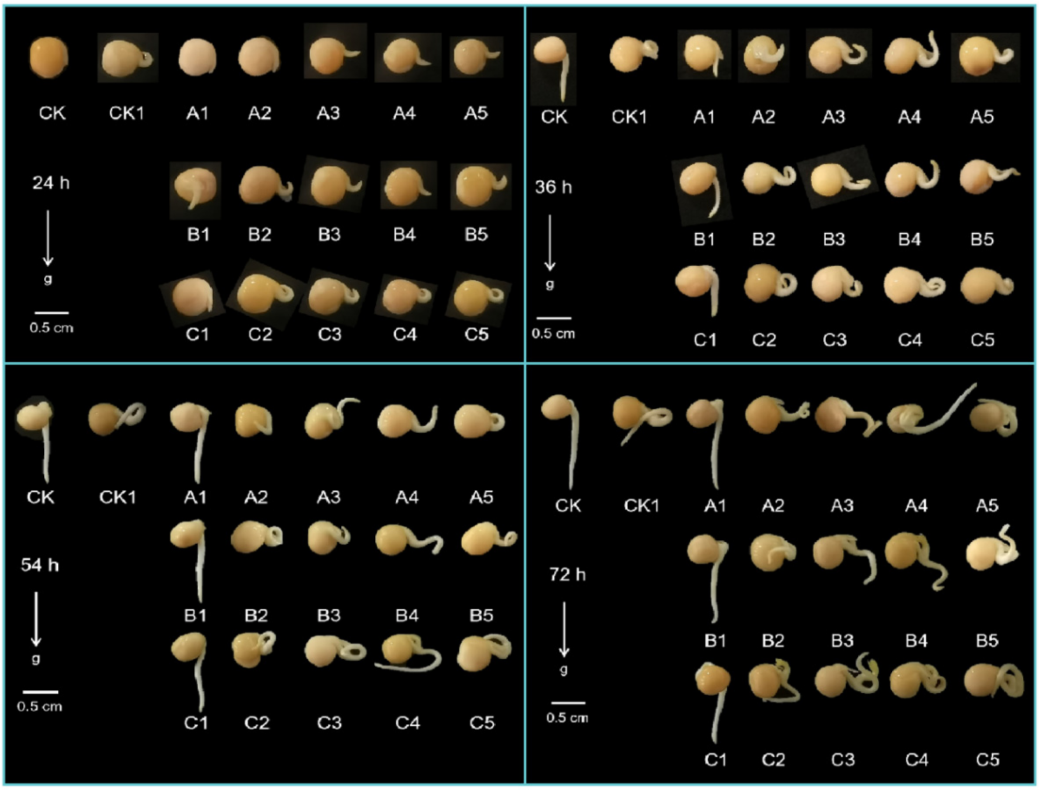

Figure S3 Growth status of pea primary roots under different treatments at 24 h, 36 h, 54 h and 72 h time intervals

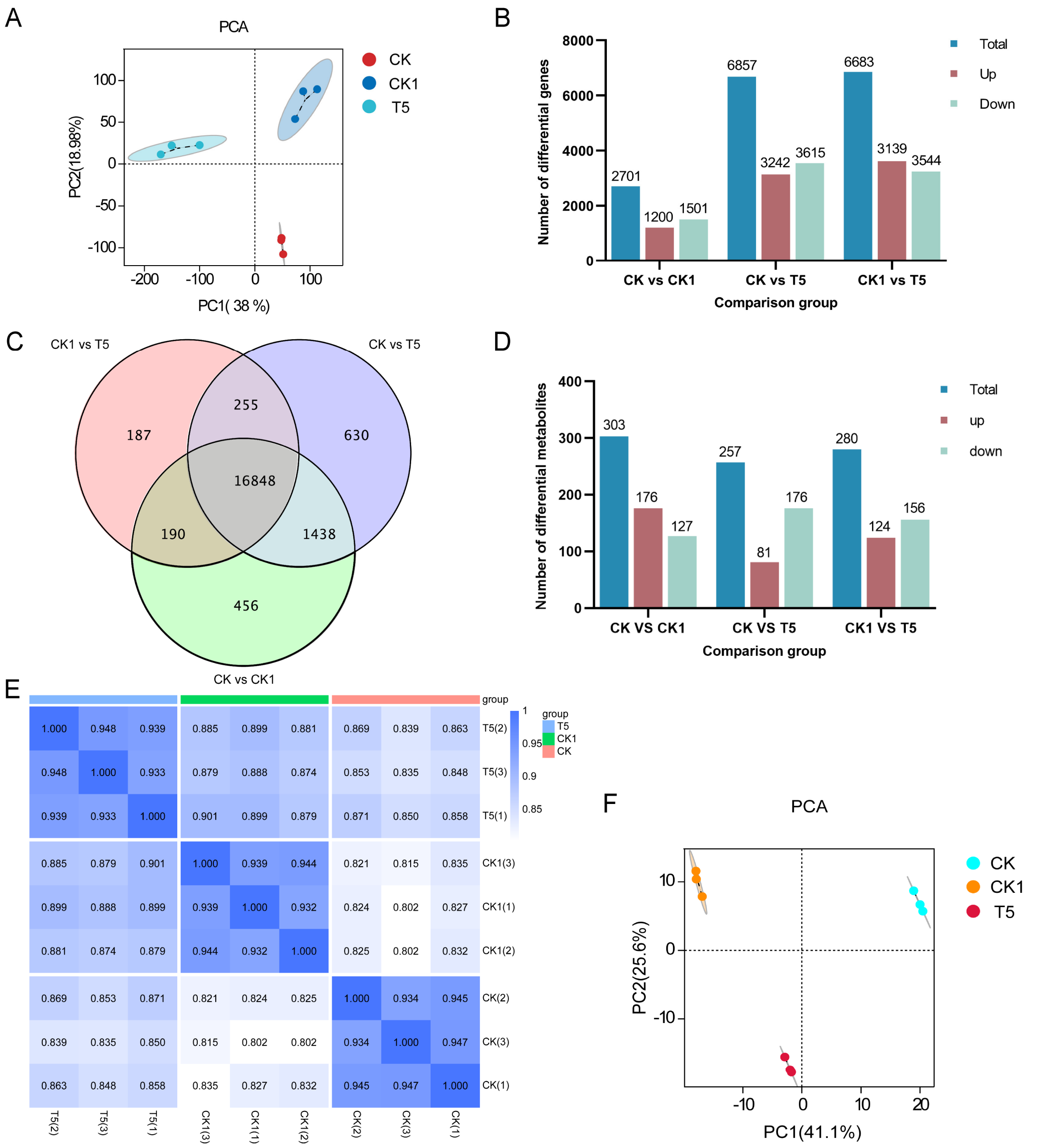

Figure S4 Overall transcriptome and metabolome analysis

(A) Principal component analysis (PCA) of differentially expressed genes (DEGs) in CK, CK1, and T5 treatment groups. (B) Number counts of DEGs. (C) Venn diagram (Venn) of DEGs. (D) Number statistics of differential accumulation metabolites (DAMs). (E) Correlation analysis of DAMs. (F) PCA analysis of DAMs. CK: water only, CK1: 150 mmol L^{-1} H_2O_2, T5: 150 mmol L^{-1} H_2O_2+10 mmol L^{-1} $CaCl_2$, the same as below.

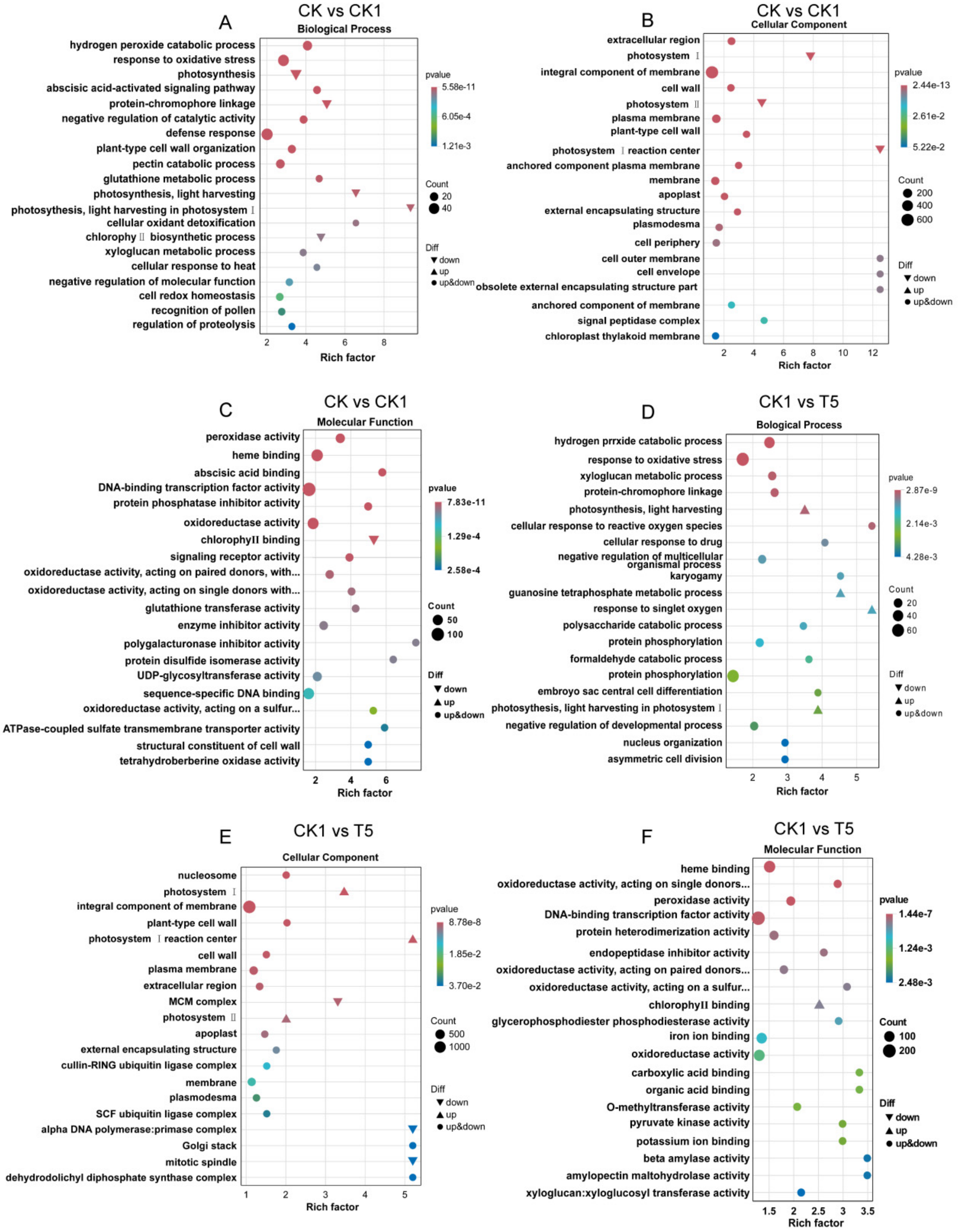

Figure S5 GO enrichment analysis of DEGs

(A), (B) and (C) are the GO enrichment results of biological processes, cell components and molecular functions of DEGs in CK VS CK1 group, respectively. (D), (E) and (F) are the GO enrichment results of biological processes, cell components and molecular functions of DEGs in CK1 VS T5 groups, respectively.

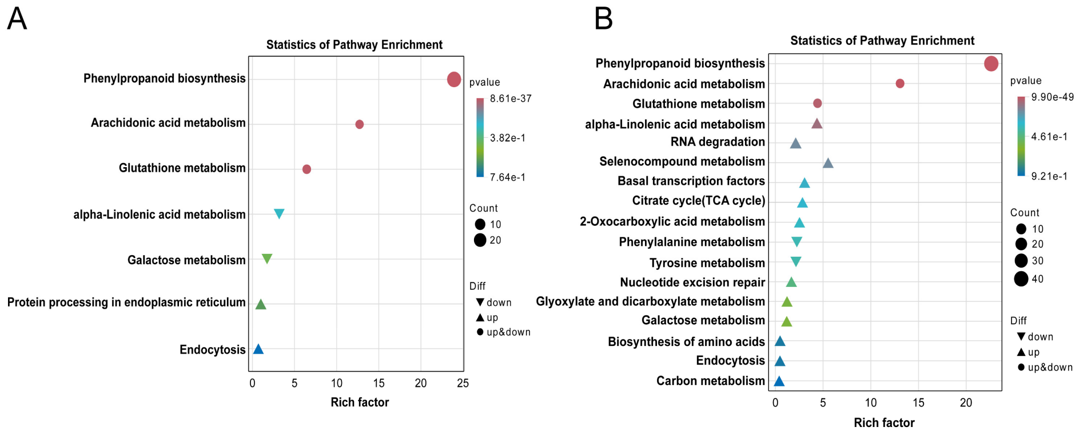

Figure S6 KEGG bubble map of transcriptome DEGs

The GO biological processes significantly enriched for "oxidative stress" (GO:0006979) and "hydrogen peroxide catabolism" (GO:004274) were then analysed for KEGG enrichment. (A) CK vs CK1; (B) CK1 vs T5.

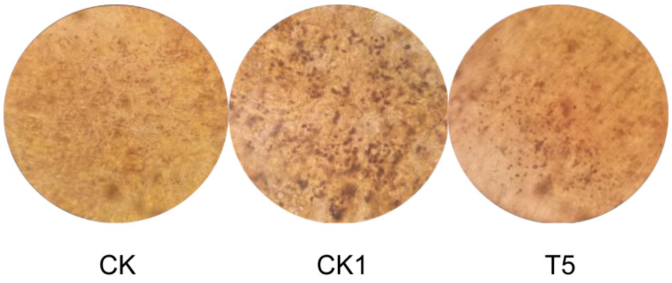

Figure S7 Starch-stained freehand sections of pea primary roots from different treatments

Pea primary roots cultured for 72 h were stained in Lugol's iodine solution for 10 min, sectioned freehand and then observed under a light microscope (16×40) for changes in starch distribution.

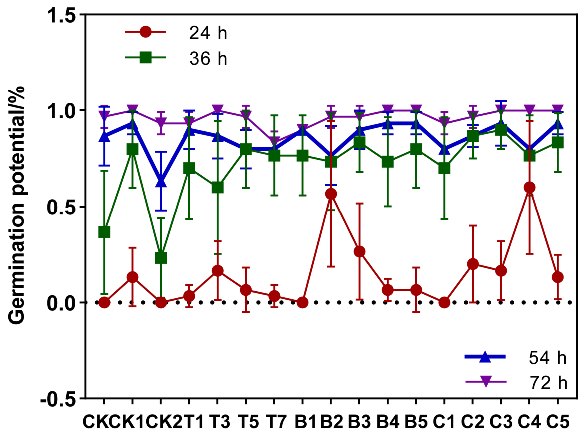

Figure S8 Pea primary root germination potential under different treatments at different times
